# Molecular Regulation and Evolution of Redox Homeostasis in Photosynthetic Machinery

**DOI:** 10.3390/antiox11112085

**Published:** 2022-10-22

**Authors:** Adeel Riaz, Fenglin Deng, Guang Chen, Wei Jiang, Qingfeng Zheng, Bisma Riaz, Michelle Mak, Fanrong Zeng, Zhong-Hua Chen

**Affiliations:** 1 Hubei Collaborative Innovation Center for Grain Industry, College of Agriculture, Yangtze University, Jingzhou 414000, China; 2 College of Agriculture and Biotechnology, Zhejiang University, Hangzhou 310058, China; 3 Department of Biotechnology, University of Okara, Okara, Punjab 56300, Pakistan; 4 School of Science, Western Sydney University, Penrith, NSW 2751, Australia; 5 Hawkesbury Institute for the Environment, Western Sydney University, Penrith, NSW 2751, Australia

**Keywords:** reactive oxygen species, signaling transduction, phylogenetic analysis, photo-protection, gene family evolution, photosynthesis

## Abstract

The recent advances in plant biology have significantly improved our understanding of reactive oxygen species (ROS) as signaling molecules in the redox regulation of complex cellular processes. In plants, free radicals and non-radicals are prevalent intra- and inter-cellular ROS, catalyzing complex metabolic processes such as photosynthesis. Photosynthesis homeostasis is maintained by thiol-based systems and antioxidative enzymes, which belong to some of the evolutionarily conserved protein families. The molecular and biological functions of redox regulation in photosynthesis are usually to balance the electron transport chain, photosystem II, photosystem I, mesophyll and bundle sheath signaling, and photo-protection regulating plant growth and productivity. Here, we review the recent progress of ROS signaling in photosynthesis. We present a comprehensive comparative bioinformatic analysis of redox regulation in evolutionary distinct photosynthetic cells. Gene expression, phylogenies, sequence alignments, and 3D protein structures in representative algal and plant species revealed conserved key features including functional domains catalyzing oxidation and reduction reactions. We then discuss the antioxidant-related ROS signaling and important pathways for achieving homeostasis of photosynthesis. Finally, we highlight the importance of plant responses to stress cues and genetic manipulation of disturbed redox status for balanced and enhanced photosynthetic efficiency and plant productivity.

## 1. Introduction

Oxygenic photosynthesis originated in cyanobacteria and subsequently the evolutionary pressure for higher redox potentials (electron source and oxygen (O_2_)) resulted in the evolution of reaction centers or photosystems. The findings that O_2_ evolving reaction center 2 (photosystem II; PSII) was originated from reaction center 1 (photosystem I; PSI) through a series of evolutionary events in algae and plants [1]. Molecular evolutionary events of photosynthesis were largely associated with genome duplication, gene fusion, and splitting, and lateral gene transfer, which drove the metabolic fluxes and photosynthetic components among diverse species of algae and plants [2]. Further, the evolution of aerobic respiration and novel biosynthesis pathways provide insights into the modern biology of complex multicellular organisms [3]. 

A fraction of captured light energy is utilized in converting water (H_2_O) and carbon dioxide (CO_2_) to glucose and O_2_ as end products [4]. The hypothesis that the first O_2_ molecules were produced as a by-product of photosynthesis is still debatable. Earlier studies proposed the appearance of O_2_ molecules as a result of the photo-dissociation of CO_2_ and H_2_O by ultraviolet (UV) radiations [5,6]. However, it has universally been accepted that cyanobacteria were the first organisms to evolve photosynthetic machinery on Earth, releasing O_2_ on a large scale into the atmosphere. Chloroplasts, the core photosynthetic organelle, have evolved with plastids from photosynthetic prokaryotes, precisely, ancestors of cyanobacteria into plants via endosymbiotic evolution more than 1 billion years ago [7]. Subsequently, the continuous oxidation of H_2_O to O_2_ has remarkably changed the redox status of the Earth, oceanic, and atmosphere. 

Reactive oxygen species (ROS) occurred soon after the first O_2_ molecules originated on Earth by the ancestors of cyanobacteria about 3.8 billion years ago [8]. Since then, these signaling molecules have been produced constantly through aerobic metabolism. It has also been proposed that ROS initiated from atmospheric oxygen soon after its release from biological systems [9,10]. Green plants (Viridiplantae) and algae produce oxygen radicals and their derivatives during aerobic photosynthesis and respiration [11]. These include free radicals [e.g., superoxide anion (O_2_^•−^), hydroxyl radical (^•^OH), hydroperoxyl radical (HO_2_^•^) and alkoxy radical (RO^•^)] and non-radical, e.g., singlet oxygen (^1^O_2_) and hydrogen peroxide [(H_2_O_2_)] molecules [12,13]. Early reports showed that ROS are toxic signaling molecules accumulated in plant cells to disturb cellular homeostasis. Overaccumulation of ROS disrupts cell metabolism which may lead to DNA damage, genome instability, and programmed cell death [14,15]. In addition, ROS are known to regulate photorespiration, growth, and stress response in plants [16,17,18]. However, our understanding regarding ROS signaling and molecular functions in cell compartments and organelles, has been limited due to technological difficulties over the last few decades [11,19,20].

As an important metabolic process, photosynthesis regulates ROS production in algae and plants. The excessive ROS as a by-product of oxygenic processes in chloroplast, mitochondria, peroxisomes, and nuclei [11,15,20,21,22,23,24], disturb photosynthetic electron transport (PET), PSII, PSI, as well as photorespiration and gene expression levels [25,26,27,28]. Photosynthetic cells have diverse layers of defense to cope with oxidative stress. The prevailing concepts show that redox signaling is carried out within antioxidative systems at the cellular level [29], and many of those associated proteins are evolutionarily conserved in plants and algae [30]. The multilayered defense systems fine-tune the ROS balance during PET, and excessive ROS diffusion and reactivity are also balanced at the organellar level throughout the life cycle [10,20,30], which is also known as redox homeostasis [31]. 

Here, we review the current knowledge of ROS homeostasis and oxygenic photosynthesis. We discuss ROS toxicity and signaling in the important organelles of a plant cell. We then analyze the molecular evolution of protein families associated with photosynthesis and redox regulation in plants and algae. In addition, we compare the evolution of ROS signaling in photosynthetic machinery in chloroplasts of distinct C3, C4, Crassulacean acid metabolism (CAM) plants and early divergent lineages of plants and algae. Finally, we highlight a few key mechanisms of how plants are adapted to high light intensities. We thus propose efficient plant biological modification of ROS homeostasis and photosynthesis to achieve better crop productivity for global food security. Also, we direct the reader to some excellent recent reviews that have focused on ROS toxicity, signaling, or photosynthesis [28,32,33,34,35,36,37].

## 2. ROS Signaling Is a Double-Edged Sword in Plant Photosynthesis

**Figure 1:**
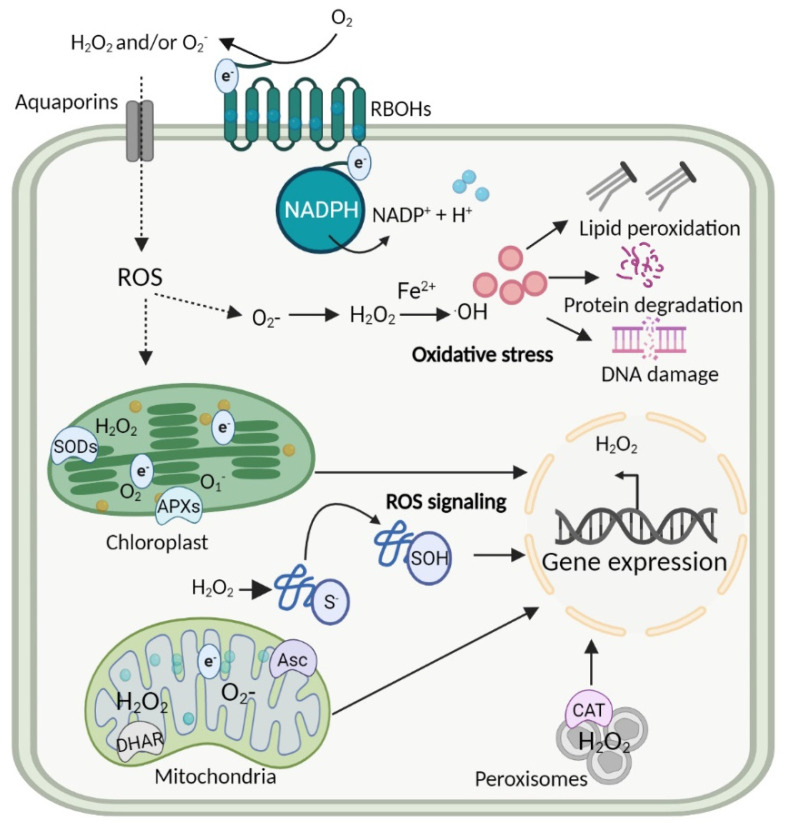
The oxidative stress signaling in plant cell compartments. ROS, such as O_2_^•−^ and H_2_O_2_ are produced by NADPH oxidases specifically RBOHs at the apoplast, accumulating in chloroplast, mitochondria, peroxisomes, and nuclei. Accumulation of H_2_O_2_ in the presence of F^2+^ form hydroxyl radicals and initiates oxidative stress that results in distorting the structure of lipids, proteins, and DNA. Later, the integration of enzymes, such as SODs, APXs, DHAR, and CAT in cell organelles maintains ROS levels and gene expression.

ROS affects almost all aspects of plant life. ROS toxicity in different species is commonly regarded as oxidative stress, potentially causing a level of damage that led to intracellular and intercellular lethality. The O_2_^•^^−^, ^•^OH, H_2_O_2_ and ^1^O_2_ reactivity may vary across or within cells, due to the site of generation, and the nature of the biomolecules. Among them, O_2_^•^^−^ and ^1^O_2_ have longer half-lives ranging from 1–4 μs with a migration distance of 30 nm in mitochondria, chloroplast, and nuclei, conversely, H_2_O_2_ is closer to 1 ms with a migration distance of 1 μm reacting with DNA and sensitive cysteine (Cys) and methionine (Met) residues. Additionally, ^•^OH’s half-life is approximately 1 ns with a migration distance of 1 nm in the Fenton reaction [37]. Furthermore, ^•^OH’s very short migration distance allows this ROS molecule to be extremely reactive with DNA, RNA, lipids, and proteins. The expression levels of ROS also vary across the subcellular organelles suggesting that the regulation of ROS is dynamic and that compartmentalization is utilized to reduce ROS lethality (Table S1). ROS signaling can be mediated by mitochondrial nicotinamide adenine dinucleotide phosphate (NADPH) oxidases, specifically, the respiratory burst oxidase homologs (RBOHs) induce ROS production in the apoplast [38,39] (Figure 1). The redox signaling complex largely consists of RBOHs, superoxide dismutases (SODs; different metal cofactors), catalases (CATs), peroxidase (PODs), glutathione peroxidase (GPXs), iron-dependent mechanisms and a network of thio- and glutaredoxins [40,41,42]. Knockout or knockdown of any of them results in modified redox signaling in plants [43].

In general, some physiological functions of plants are regulated via the interaction of ROS with Cys and Met residues of key proteins [44,45]. The oxidative posttranslational modifications (oxiPTM) oxidize the residues at physiological pH, thus altering the structure and functions of proteins [44,45]. Therefore, the passive ROS diffusion through aquaporins and the direct interaction occurring in chloroplasts and nuclei should be tightly controlled by antioxidant systems to neutralize ROS and oxidation to mitigate damage [46,47]. For example, H_2_O_2_ profiling analyses showed that it catalyzes the biochemical reactions at lower concentration (e.g., average H_2_O_2_ concentration, 10 μM in cellular compartments especially peroxisomes and apoplast mediate normal cell division) but creates oxidative damage at higher concentrations in plants (Supplementary Figure S1) [48,49]. The H_2_O_2_ scavenging is accomplished by coordinated activities of antioxidative enzymes [50] such as SODs, which processes O_2_^•^^−^ to H_2_O_2_ and are ubiquitously expressed in apoplast, cytosol, peroxisomes, chloroplasts, mitochondria and nuclei [51,52].

Photosynthesis in chloroplasts is directly linked to cellular redox regulation in plants [16]. Chloroplast-associated ^1^O_2_ is produced from chloroplast triplet state (^3^Chl) by interacting with O_2_ molecules (ground state ^3^O_2_), specifically in PSII under varying irradiance [49,53]. Elevated ^1^O_2_ levels in the PSII reaction center cause photo-inhibition resulting in oxidative damage [54] and metabolic breakdown, leading to oxidation of the D1 protein, which specifically dephosphorylates the PSII reaction center [55]. The irreversible photo-inhibition drives the expression of ^1^O_2_ related genes, which activate the antioxidative system, and ultimately induce resistance against high light (HL) and other stresses in plants [54,56,57]. Moreover, PSI oxidation is known as the Mehler reaction—photo-reduction of O_2_ [58]. This reaction is catalyzed by thylakoid and stromal-associated SODs, producing O_2_^•−^ as the first product and then dismutase O_2_^•^^−^ to H_2_O_2_. H_2_O_2_ may cause oxidation to Calvin-Benson Cycle (CBC) components regulated by the thioredoxin system [59], 2-Cys peroxiredoxin and ascorbate peroxidase (APX) [60]. Further reduction of H_2_O_2_ to H_2_O is catalyzed by the integration of APX, thiol related enzymes such as thioredoxin (TRX), peroxiredoxin (PRX) and NADPH. However, the enzymatic system is tightly regulated in cellular compartments where H_2_O_2_ retains the potential to move out of chloroplast and mitochondria interacting with transcription factors for retrograde signaling [56,61,62,63,64], which regulate the gene expression in the nucleus. In Arabidopsis, the NAC domain containing protein 17 (ANAC017) interacts with enhanced H_2_O_2_ levels and modulates the gene expression [65]. The ANAC017 activity is inhibited by radical-induced cell death 1 (RCD1), which mediates ROS-related retrograde signaling in mitochondria has also been anticipated [66]. Examining the dynamics of chloroplast demonstrate that this organelle can actively sense environmental cues, regulating the nucleus-chloroplast communication and gene expression. Chloroplast associated ROS retrograde signaling is largely dependent on ^1^O_2_, produced as a by-product of PSII reactions. The production of ^1^O_2_ facilitates the chloroplast to nuclear communication, which ultimately modulates the gene expression, responds to stress, and programmed cell death [67]. Taken together, these studies provide a mechanism for how PSII and PSI mediate redox reactions are the keys to balance photosynthesis.

Both PSII and PSI are sensitive to light intensities and other abiotic stresses. Redox homeostasis is disturbed when the rate of damage is higher than repair, causing photoinhibition [68]. The excessive energy under HL can be dissipated as heat via non-photochemical quenching (NPQ), which subsequently adjust the chloroplasts composition and metabolism. The presence of an antioxidant system in chloroplast helps in nucleus-chloroplast communication that drives the gene expression. In addition, peroxisomes accumulate H_2_O_2_ under photorespiration [49], which is removed by peroxisome associated CATs [69]. Recent findings indicate that antioxidants ascorbate (Asc) and glutathione (GSH) regulate gene expression under stresses [70,71], suggesting their dynamic nature and high sensitivity to stress cues. In summary the mechanisms are now well-defined, reveal that moderate levels of ROS are essential for cell proliferation, photosynthesis mechanisms, and maintaining redox homeostasis at a basal level within chloroplasts. However, ROS signaling components are less studied in relation to the evolution of the key protein families from algae to plants.

## 3. Molecular Evolution of Redox Regulatory Network

Modern chloroplasts arise from photosynthetic prokaryotes. Green plant chloroplast genomes contain small proportions of genes than their ancestors [7], indicating that endosymbiosis resulted in the loss and relocation of genetic information. During the transition from aquatic life to terrestrial habitats, green plants have evolved some new pathways for redox regulation and defense systems. Trx-based redox regulation has been observed in all groups of life, indicating a significant role under diverse redox environments [72,73]. 

**Figure 2:**
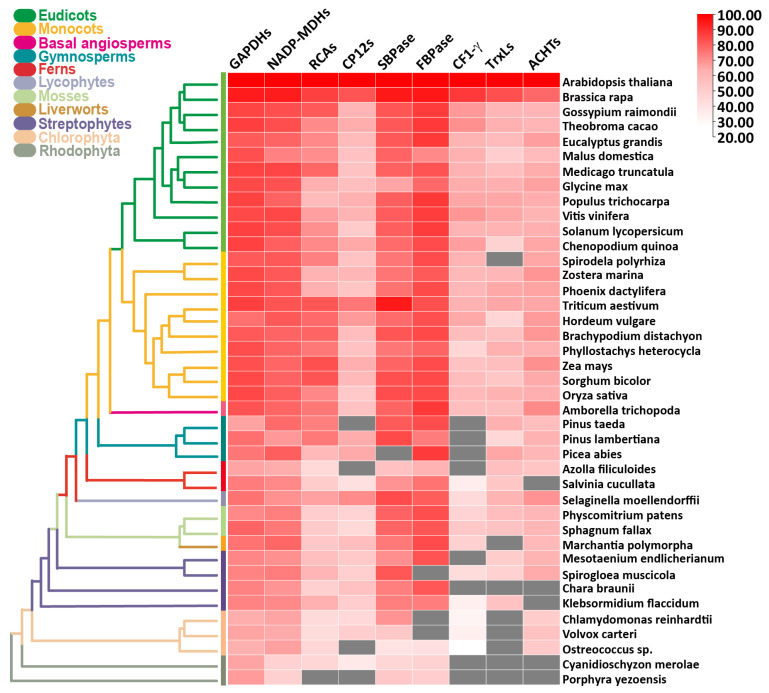
Comparative molecular evolution of key photosynthetic redox regulators and photosynthesis protein families in algae and land plants candidate proteins were extracted using BLASTP (NCBI), and the all-against-all BLASTP search with satisfied E-value of 10^−10^ and query coverage of 50%. Heat map was generated using TBtools [85] from the data of seven photosynthesis-related proteins and four redox-related families. Black squares indicate the proteins that do not satisfy the selection criteria. ACHTs: atypical Cys His-rich, CF1-y: gamma subunit of ATP synthase; CP12; FBPase: fructose-1,6-bisphosphatase; GADPH: glyceraldehyde-3-phosphate dehydrogenase; NADP-MDH: NADP^+^ dependent malate dehydrogenase; RCA: ribulose-1,5-bisphosphate carboxylase/oxygenase activase; SBPase: sedoheptulose-1,7-bisphosphatase; TrxLs: Trx-like.

**Figure 3:**
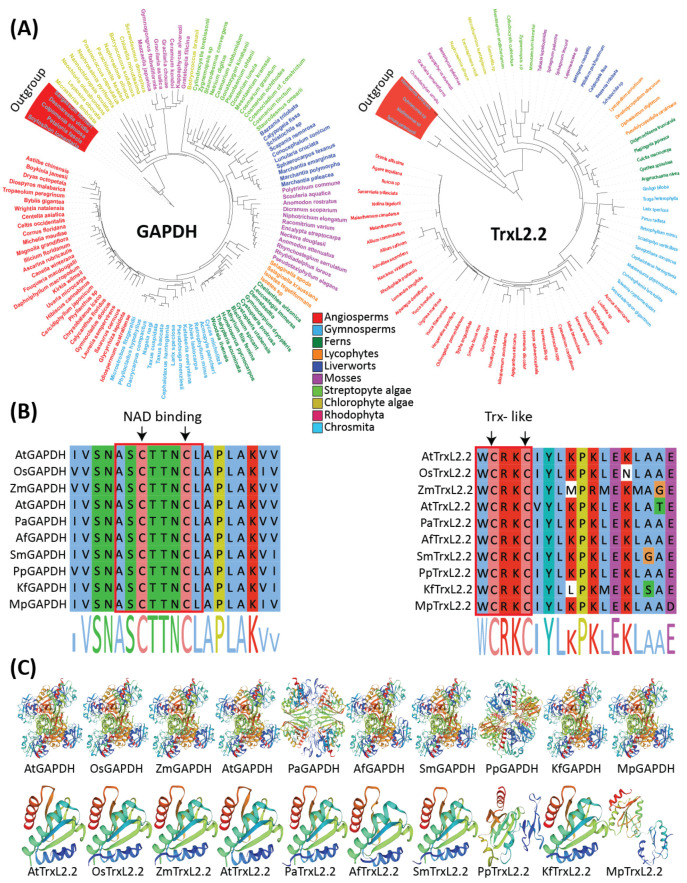
Phylogenetic, conserved domains and 3D protein analysis of GAPDH and TxL2.2 in Plants and algae (**A**) Phylogenetic tree of GAPDH and TrxL2.2 from representative plant and algae lineages. The alignment was performed using MAFFT v7.409 [86]. A maximum likelihood tree was generated using RAxML-HPC2 on XSEDE. (**B**) Sequence alignment of GAPDH and TrxL2.2 for conserved NAD binding and Trx-like domains, respectively. The alignment was performed using Jalview [87] with default parameters. (**C**) 3D protein structures were predicted using a Swiss-prot server in representative algae and plant species. At, *Arabidopsis thaliana*, Os*, Oryza sative;* Zm*, Zea mays;* Amt*, Amborella trichopoda;* Pa*, Picea abies;* Af*, Azolla filiculoids;* Sm*, Selaginella moellendorffii;* Pp*, Physcomitrella patens;* Kf*, Klebsormidium flaccidum;* Mp*, Marchantia polymorpha*.

The alterations in the environment cause large evolutionary pressure, leading to the emergence of new functions to existing genes and the formation of new genes [74,75]. Comparative genomic analyses revealed the regulatory roles of conserved regions of key gene families for diverse cellular functions such as photosynthesis in eukaryotes [76]. Thus, the evolutionary histories of redox homeostasis and photosynthesis can be drawn through comparative genetic analysis, gene expression profiles, phylogenies, conserved domain analysis, and prediction of 3D protein structures. In our previous comparative molecular evolution studies, we have revealed conserved features of over 100 gene families in green algae and land plants [77,78,79,80,81,82,83,84]. Here, the functional regulatory networks among distinct species were analyzed through comparative genetic analysis of sequences in redox signaling of photosynthesis from evolutionarily important lineages of plants and algae (Figure 2 and Figure 3, Supplementary Figures S2–S5).

## 4. ROS Related Gene Families Are Highly Conserved across Land Plants and Green Algae

We found that there are evolutionarily conserved features for ROS signaling and photosynthesis in the examined major green plant lineages (Figure 2, Supplementary Figure S3). Phylogenetic analysis suggests that these families may have evolved from streptophyta. The structure of TrxLs contains conserved WCRKC domain with two cysteine residues which serve as redox switches. 

The dominant regulators with oxidation activities in chloroplast are Trx-like, NADPH-dependent Trx- reductase C (NTRC) proteins [88]. These proteins further comprise Trx-like 2 (TrxL2), and atypical Cys His-rich Trx (ACHT) groups based on oxidation factors [89,90,91,92,93,94,95], and catalyze H_2_O_2_ reduction through interacting 2-Cys PRX (2CP). Different subfamilies of Trxs may exist, such as Trx-x in Arabidopsis [96], Trx-y in green algae *Chlamydomonas reinhardtii* and Trx-z in Arabidopsis and *Solanum lycopersicum* [97,98]. The Trx- gene number in plant species is largely expanded in angiosperms, for example, there are more than twenty Trx isoforms in Arabidopsis [99]. The oxidation factors family proteins can be further classified into Trx-like *f-, m-, x-, y-, or z-* with unknown functions except for TRX-*f*, which specifically carried out the oxidation of target photosynthesis proteins [93,100]. In addition, five isoforms of ACHT were reported in Arabidopsis [101], concomitantly ACHT1 and ACHT2 revealed an association with targeted oxidation. 

Recent in vivo experiments confirmed the roles of Trx and ACHT in oxidation processes in the chloroplast [102]. Yokochi et al. [102] discovered that the Trx-*f* and TrxL2.1 serve as oxidation factors of CBC enzymes, ribulose-1,5-bisphosphate carboxylase/oxygenase activase (RCA) and gamma subunit of ATP synthase (CF1-γ), respectively ACHT1 and ACHT2 play a redundant role in oxidizing fructose-1,6-bisphosphatase (FBPase) while phosphoribulokinase (PRK) regulation under Trx- system is still elusive. Comparative genomic studies suggest that TrxL2.1 and TrxL2.2 are conserved in photosynthetic organisms including flowering plants, mosses, and streptophyte algae (Figure 2 and Figure 3), but not in chlorophyte algae such as *Chlamydomonas reinhardtii* [88]. Both TrxL2.1 and TrxL2.2 share functional residues and biochemical features but differ in expression patterns [90]. In Arabidopsis, TrxL2.1 and ACHT2 highly express in leaves [102,103]. The *trxl2.1* and *acht* knockout mutants displayed similar phenotypes and physiology (chlorophyll content and photosynthetic parameters) compared to those in the wild type under low light. In contrast, *ntrc* mutants showed reduced ROS levels with reduced growth and pale green phenotypes. Interestingly, *ntrc* mutants with background Trxl2.1 largely recovered the wild-type phenotype, suggesting that 2CP retains the potential to accept electrons from both TrxL2.1 and ACHT, and the NTRC oxidation system. In addition, it was revealed that reduced growth of Arabidopsis *ntrc* mutants was associated with overoxidation of CBC proteins rather than imbalanced redox regulation or reduced H_2_O_2_ scavenging. A remarkable positive association was observed between ACHT levels and NPQ, which reveals overexpressed ACHT led to high NPQ [102]. These studies suggest that TRX and ACHT are key players in redox regulation in CBC. Future experiments will uncover the underlying mechanism of remaining redox regulators for photosynthesis.

## 5. Key Photosynthesis Related Gene Families Are Evolved from Streptophyta Algae

We found that CBC enzymes share common features among all tested evolutionary lineages (Figure 2, Supplementary Figure S4). Phylogenetic analysis suggests that these families may have evolved within streptophyta. For instance, the structure of GAPDH holds a conserved functional NAD binding domain that is used as a coenzyme.

The CBC enzymes including RCA, glyceraldehyde-3-phosphate dehydrogenase (GAPDH), CF1-γ, FBPase, SBPase, and PRK [59,73] are highly sensitive to irradiance with high activities under light conditions. RCA is involved in the activation of Rubisco and belongs to the AAA+ family [104], which contains the α and β isoforms. These two isoforms may be generated from alternate splicing or encoded by different genes [105] in green algae and land plants with the exception few species including Chlamydomonas have only a short RCAβ isoform [106]. Both RCA isoforms differ with the presence of two conserved C-terminal Cys residues, where reduction is catalyzed by Trx*f* and ATP/ADP ratio under light conditions. GAPDH is further grouped into *gapA* and *gapB*, sharing similar composition with a distinction C-terminal extension residing 30 amino acids (aa) in *gapB*. These extended aa contain Trx- target Cys residues regulating light and dark reactions. *gapA* and *gapB* are present in streptophyta and green algae [107,108], with the exception that *gapB* was found absent among most oxygenic phototrophs, containing only *gapA* copies or additional *gapC* [109].

FBPase is required for dephosphorylation of fructose-1,6-bisphosphate (F1,6P) to fructose-6-phosphate (F6P). Light activation of FBPase and PRK was first discovered in Chlorella [110] and subsequently observed in higher plants [111,112]. PRKs show striking features along the evolutionary lineages, for example, homodimeric in eukaryotes, heterodimeric in cyanobacteria, and octameric in non-photosynthetic prokaryotes [113]. Considering the redox regulatory components, FBPase and PRK activities are largely dependent on Trx*f* and Trx-*m*, respectively, which carry out disulfide reduction and make them activated [114]. As an oxidizing enzyme, SBPase catalyzes sedoheptulose-1,7-bisphosphate (S1,7P) to sedoheptulose-7-phosphate (S7P), which has been discovered in many photosynthetic organisms except cyanobacteria. However, cyanobacteria contain a bifunctional SBPase with similar activities [115]. The findings that share sequence similarity with SBPase and Trx*f* indicate that both enzymes may have a common evolutionary origin [116]. 

C4 and CAM photosynthesis metabolism is regulated by NADP^+^-dependent malate dehydrogenase (NADP-MDH). NADPH serves as an electron donor during C4 CO_2_ fixation by reducing oxaloacetate into malate during the day and at night in CAM plants [117]. The molecular mechanism associated with NADP-MDH was first observed in the C4 plant, *Sorghum bicolor*, and subsequently in C3 and CAM plants [118]. NAPH-MDH is highly dependent on light-responsive Trxs, possibly Trx*f* or Trx*m* types evolved from *Chlamydomonas reinhardtii* [119]. In plants, NADP-MDH reside Cys extensions both at the C- and N- terminals, but green algae contains only C-terminal residues [119]. Although enzymes associated attributes have been reported, functional validations in a range of evolutionarily important green plants are yet to be conducted in future research work.

## 6. Antioxidant Related Gene Families Are Conserved across Land Plants and Green Algae

In our previous publications, we reported an early evolution of antioxidative enzymes from Chlorophyte algae [63,78]. SOD, CAT, POD, GPX, GR, glutathione S-transferase (GST), ascorbate peroxidase (APX), monohydroascorbate reductase (MDHAR) and dehydroascorbate reductase (DHAR), Asc, GSH, and antioxidant, phenolic compounds, alkaloids, flavonoids, carotenoids, free amino acid, and alpha tocopherols [40,41,42] play crucial roles in catalyzing oxidation-reduction reactions to regulate redox homeostasis in plants. The first reported antioxidant enzyme was SOD, which removes ROS, and is thought to have evolved even before the origin of eubacteria and archaea [30]. In cyanobacteria, several antioxidants containing protein and non-protein molecules have evolved from *Synechococcus* PCC [120]. The constant release of O_2_^•−^ anions from the photosynthetic machinery and respiratory electron transport chain [120], and on other cellular surfaces [121] are scavenged by SODs and superoxide reductase (SORs). Similarly, PODs and CATs potentially reduce the stability of H_2_O_2_, RO^•^ and ^•^OH radicals [122]. Although O_2_^•-^ are produced as by-products of aerobic life, they also promote iron acquisition, cell signaling, and growth [121,123]. O_2_^•−^ appears to function differently upon over-accumulation in plant cells, destabilizing DNA by inducing Fenton reactants [124] and depletion of 4Fe-4S clusters in proteins [125], which are required for photosynthesis and amino acid biosynthesis, respectively. Taken together, the results provide a clue that redox homeostasis needs to be maintained to regulate both the molecular and biological functions of organisms. This study highly supports the existence of SODs in the genomes of Eukarya, e.g., land plants and green algae (Supplementary Figure S5A,B), archaea, and bacteria [126]. 

## 7. Redox Regulation during Photosynthesis

The light-driven electron transport chain (ETC) mediates its function by recruiting H_2_O, generates reducing power NADPH in the thylakoid membrane, and initiates the proton (H^+^) gradient that leads to ATP synthesis [3]. In chloroplasts, redox status is regulated by Cys residues of photosynthetic enzymes, which are highly conserved and retain the potential to switch ON or switch OFF signaling [127], indicating some fine-tuned mechanisms are involved during photosynthesis. The dominant ROS that reduces photosynthetic efficiency are ^1^O_2_, H_2_O_2_ and O_2_^•−^, in PSI [128]. Plant chloroplasts have evolved different strategies to minimize photo-oxidative stress. One such strategy includes the maintenance of CBC enzymes that contain redox switches as Cys residues [59,129]. Other strategies involve non-cyclic electron flow (NCEF, reduced photochemical energy; PQ and ETC), and cyclic electron flow (CEF), photorespiration, balanced PSI/PSII ratio, O_2_ photo-reduction, and protein quality have been adopted to increase photosynthetic efficiency [130,131,132,133]. Lowering PQ and ETC initiates NPQ which results in subsequent reductions in the excitation energy of chlorophyll *a*, which is then dissipated as heat [133,134,135]. On the other hand, CEF produces higher ATP relative to NADPH; thus O_2_^•−^ and H_2_O_2_ become the acceptors of energy in PSI [136]. CEF seems to be important because it reduces ^1^O_2_ synthesis at PSII and increases the proton gradient in thylakoid membrane and activates NPQ processes [137] (Supplementary Figure S6). Whether the cyclic or non-cyclic mechanism activates to protect the photosynthetic machinery is highly dependent on photosynthetic cell and/or organelle types and environmental cues. 

## 8. Regulation in Calvin Benson Cycle

**Figure 4:**
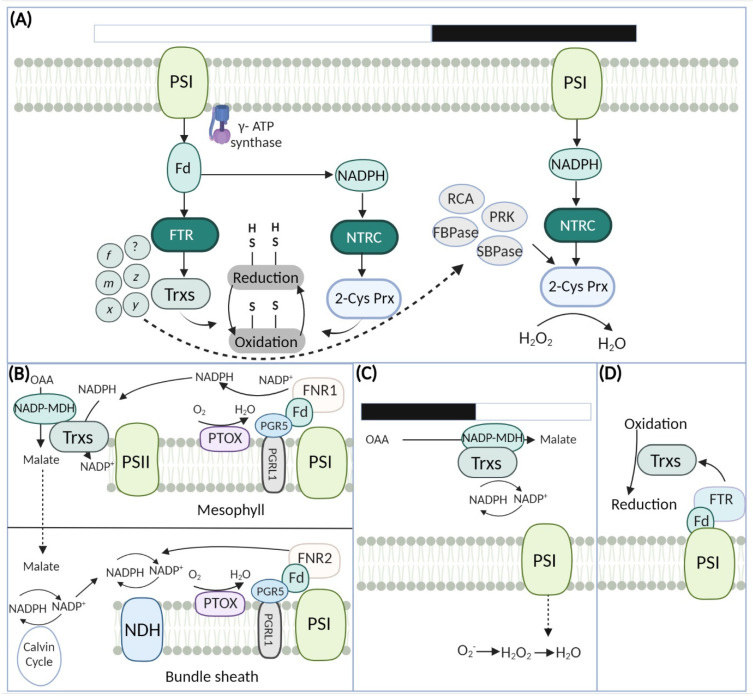
Redox signaling in evolutionary distinct photosynthetic machinery. Light and dark periods activate and deactivate the target photosynthesis enzymes. (**A**) Calvin-Benson Cycle (**B**) Kranz anatomy (**C**) CAM photosynthesis (**D**) Early divergent land plants and green algae. Trx-like proteins carry oxidation and reduction reactions to maintain PSI homeostasis. RCA: ribulose-1,5-bisphosphate carboxylase/oxygenase activase; FBPase: fructose-1,6-bisphosphatase; SBPase: sedoheptulose-1,7-bisphosphatase; PRK: phosphoribulokinase; PSI: photosystem I; PQ: plastoquinone, PTOX: plastid terminal oxidase; PGR5-PGRL1: 5-PGR5-like photosynthetic phenotype 1; FNR: ferredoxin; NADPH reductase; Fd: ferredoxin.

Here, we propose a simplified model of the mechanisms of conserved enzymes in the operation of photosynthesis (Figure 4A). Light and dark reactions are catalyzed by unique regulatory pathways involving enzymes with conserved functional Cys residues [59,129]. Among them, Trx- and NTRC systems play a significant role in maintaining redox homeostasis in the chloroplast [73]. As well, Trx- system encoding multicomponent may be localized in chloroplast, mitochondria, nucleus, and cytoplasm [99], thus plant chloroplasts have widely adapted Trx- system that carried out CO_2_ fixation via disulfide reduction of CBC enzymes, including RCA, CF1-γ, FBPase, SBPase and PRK [59,73]. Increasing evidence suggest that regulatory proteins become higher in expression under light, while lower in the dark reactions [138,139]. The reducing powers from ETC are transferred to TRX through ferredoxin (fd) and ferredoxin-TRX reductase (FTR) [140]. Further, the activated TRX targets the corresponding photosynthetic enzymes, and reduce the disulfide bond. Also, it was found that NTRC power transfer efficiency is higher than TRX proteins; thus, NTRC may function as main regulator donating an electron to 2CP. In plants, the affected stability of NTRC led to phenotypic abnormalities, decreased chlorophyll content, and higher NPQ [141,142,143,144,145,146,147]. 

Under dark reactions in the chloroplast stroma, the redox-related proteins are reoxidized and reoxidation is needed to balance the NADPH status in the chloroplast, especially under light fluctuations. This phenomenon is accomplished by a plastic malate valve, consisting of oxaloacetate/malate transporter 1 (OMT1) [148] and malate dehydrogenases (MDHs), which carry out stromal OAA reduction to malate [149]. Coexisting MDHs isoforms have a differential preference for coenzymes, such as NADPH-MDH has a strong preference for NADP [H] [150] while NAD-MDH chooses NAD [H] [151]. Interestingly, their co-existence has caused much confusion among scientists for several years. The opinion is that chloroplasts use NAD, merely converting it into NADP by NAD kinase 2 (NADK2) under light, while NADH is not further phosphorylated [152]. Earlier studies showed that FNR’s have strong affiliations for NADP [H] [153,154,155]. Similarly, the chloroplast stromal NADPH conversion into NADH interferes with balanced NADP [H] synthesis and utilization and thus affects photosynthesis, precisely CBC [156]. A recent study revealed that NADP-MDH plays a crucial role in chloroplast redox homeostasis under varying light intensities [127]. This pathway activates chloroplast NADP^+^ dependent MDH via Trxs, subsequently, the generation of NADP^+^ and increased reducing power is transported and discharge pressure from the chloroplast. The loss function of MDH in Arabidopsis leads to stunted growth in response to short days or fluctuating light while in the dark the phenotypic differences are still unchanged. MDH-mediated redox regulation plays a crucial role in response to prolonged dark period and varying light intensities [127]. Considering the importance of oxidation, the identification of uncharacterized players will further explore the associated pathways for redox homeostasis in photosynthesis.

The core photosynthetic enzymes have also been found in microorganisms [157,158]. The question arises, how redox switches are regulated by nonplastidial type homologs of CBC enzymes? It should be noted that these homologs do not hold redox switches while remaining activated without reduction via redox transmitters. Dark conditions are essential for maintaining redox status, such as FBPase restricts excessive ATP utilization during the reaction catalyzed by phosphofructokinase [73]. In addition, ATP synthase restricts a useless reverse reaction and ATP hydrolysis [159,160] and MDH limits the reducing molecules’ export to chloroplasts through the malate valve [149,161,162]. The structure of MDH contains Cys residues both at N- and C- terminals indicating that dark mediated MDH regulation is tightly controlled [161,163]. While FBPase and MDH have essential functions in chloroplast metabolic homeostasis under varied light intensity and metabolic states [139,164]. the detailed mechanism of redox regulation for plant metabolism is still elusive. 

## 9. Regulation in Kranz Anatomy

C4 photosynthesis appears in two types of cells [mesophyll cell (MC) and bundle sheath cell (BSC)], known as Kranz anatomy (Figure 4B). It utilizes an additional two ATPs and results in a higher photosynthetic rate, lower photo-respiratory flux [165] and reduced CO_2_ compensation point [166]. The transition of photorespiration between both cell types upregulates uncoupling protein (UCP) and bundle sheath mitochondria alternative oxidase (mAOX), which serve as a valve to scavenge NADH via glycine carboxylase mediated mechanism [166]. The activated carboxylation in C4 bundle sheath export NADH to the respiration electron chain, and AOX maintain the ROS balance [166]. Earlier studies showed no significant alterations in the redox systems of C4 and C3 (comprising only MC). The differences indicate the use of alternative decarboxylation enzymes in distinct species [165,167].

In general, C4 species employ CEF around PSI, export electron from Fd to plastoquinone (PQ) and generates an H^+^ gradient in the thylakoid membrane. That the outcome is a loss of PSII activity, and subsequent accumulation of PSI subunits in chloroplasts [168,169], which may involve the 5-PGR5-like photosynthetic phenotype 1 (PGR5-PGRL1) pathway [170,171]. This pathway diverts the excessive flux via plastid terminal oxidase, namely PTOX or IMMUTANS, and discharges pressure from chloroplast [172]. These differences indicate that some aspects of redox homeostasis in C4 photosynthetic machinery may have resulted from divergent evolutions from C3 to C4 in angiosperms. In addition, the carboxylation pathway may differ with the prevalence of CEF between MC and BSC and the CEF/NCEF ratio in plant species [173,174]. Looking forward, studying the regulation of redox status between photosynthetic cycles and redox distribution between MC and BSC and other associated cycles will uncover the regulation underlying redox homeostasis in C4 plants. 

## 10. Regulation in Crassulacean Acid Metabolism (CAM)

Crassulacean acid metabolism (CAM) functions by closing stomata during the daytime and fixing CO_2_ at nighttime [175,176] (Figure 4C). CO_2_ fixation occurs in malic acid at night, which is subsequently stored in the vacuole of MC. Similar to C4 photosynthesis, NADP-MDH dependent oxidation linked with Trx systems has been observed in CAM plants [117]. In C3 angiosperms, over-reduction of acceptor side of PSI and chloroplast stroma mediated “match valve” system transfer reducing equivalents to the cytosol, which generates NADP^+^ under light fluctuations [163,177]. It is still largely unknown whether CAM plants have a water-water cycle as C3 plants for adjusting PS1 redox status or restricted CO_2_ fixation under malic acid exhaustion. Recently, studies based on redox regulation in CAM photosynthesis of *Bryophyllum pinnatum* [178], *Dendrobium officinale* [179,180], and *Vanilla planifolia* [181] demonstrate that CEF and water-water cycle could be an important regulator as adjusting PSI redox homeostasis under light fluctuations. However, research work on redox homeostasis and photosynthesis in CAM plants requires acceleration to better compare the research advances in C3 and C4 plants. 

## 11. Regulation of Redox in Early Divergent Plants and Green Algae

Green plants acquired the potential of oxygenic photosynthesis, which produces ATP and NADPH for CO_2_ fixation and subsequent pathways. Dominant Trx-based redox regulation is important in all groups of life under diverse redox environments. The transition of plants from an aquatic environment to land may have enabled the evolutionary innovation of new pathways or new components of pathways for oxidative regulation and defense systems. Considering the redox regulation in oxygenic photosynthetic organisms, the FTR mechanism appeared dominant [157] (Figure 4D). It has been hypothesized that plant NTRC functional pathways resemble cyanobacteria FTR and are directly associated with metabolism in response to light [182,183]. However, future research work is required to test these hypotheses experimentally. 

## 12. Metabolism of Redox Regulators in Maintaining Redox Homeostasis for Photosynthesis

The detoxification of ROS in chloroplast and associated organelles during photosynthesis is carried out via complex pathways. For instance, the ascorbate pathway plays crucial roles in diverse photosynthetic processes with ascorbate-dependent and independent routes. Ascorbate is produced in mitochondria and utilized in the chloroplast, whereby the transportation to chloroplast membranes from cytosol occurs via ascorbate transporters and stabilizes the thylakoid membrane against oxidation by reducing tocopheroxyl radicals to non-oxidizing form [184]. In the ascorbate-dependent pathway, oxidation of H_2_O occurs due to damage to PSII [173]. Later, the integration and interaction of antioxidative enzymes reduce H_2_O_2_ to water. On the other hand, the ascorbate-independent cycle is regulated via Fd/Fd-dependent Trx reductase (FTR)/TRX or NADPH/NADPH Trx C (NTRC) and PRX [185]. It was revealed that more than one type of ascorbate transporters may exist, which facilitates the ascorbate diffusion from cytosol to chloroplast stroma [186,187]. One such group includes nucleobase- ascorbate transporters (NATs) or nucleobase- cation symporter 2 (NCS2) comprising ascorbate transportation and DNA bases [188]. They have been found in all kingdoms of life ranging from unicellular bacteria to multicellular animals [188,189]. All members in the NAT family share similar features regarding amino acid sequences and conserved trans-membrane proteins [190,191]. Functional characterization of NAT proteins in plants figures out their vital roles in plant growth and development. Taking an example, leaf permease 1 (*Lpe1*) was first identified as a NAT family member in plants [192], which helps in the transport of xanthine and uric acid but not ascorbic acid [191]. Loss function of *Lpe1* phenotype shows defective chloroplasts and lost plasma membrane integrity [192]. Other group includes phosphate transporter proteins, such as Arabidopsis AtPHT4.4 is a member of the phosphate transporter 4 family that facilitates ascorbate movement into chloroplast envelope membranes [193]. AtPHT4;4 serves as a co-transporter with Na^+^/P_i_ and Cl^−^ dependent activity facilitating the Δψ to become a driving force. The loss function of *AtPHT4;4* reduces ascorbate levels and disrupts the xanthophyll cycle, which is ultimately responsible for removing excessive photosynthetic energy as heat [193]. Remarkably, the loss of *AtPHT4.4* does not affect shoot phenotype and shows HL tolerance when its antioxidative properties are most required. In addition, *AtPHT4.4* is localized in palisade cells rather than MC, which calls for further investigations. 

The cellular functions and comparison of antioxidants have been investigated in model and non-model C3 and C4 plants, such as, *Helianthus annuus* (C3) and *Sorghum bicolor* (C4) [194], *Triticum aestivum* (C3) and *Zea mays* (C4) [195,196], *Cleome spinosa* (C3) and *Cleome gynandra* (C4) [197], and *Flaveria* sp. [198]. *Flaveria* sp. had higher redox scavenging for CATs under C3 while *APXs* and *GRs* were found up-regulated in C4 photosynthesis [198]. CATs activities were inhibited in a C4 species *Flaveria bidentis,* which were later confirmed at the protein level [198], suggesting the change in photorespiration may result in altered antioxidant activity. Similarly, APXs-associated scavenging was found to be lower in C4 when compared to C3 species [197,199]. Taking the example of *Flaveria bidentis*, these changes occur from young leaves (C3) to mature plants (C4) due to chloroplasts’ dimorphic nature [200]. Furthermore, APX-mediated H_2_O_2_ scavenging was down-regulated four-fold, while PRXs were up-regulated five-fold [199]. Their results provide a potential mechanism for antioxidant activity in both types of photosynthesis, which may be activated vice versa depending on chloroplast responses. Interestingly, both APX and PRX work in coordination; for instance, any genetic change in one will be compensated by the other enzyme to release ROS from chloroplast [201,202]. Considering the role of antioxidative enzymes in redox regulation, we anticipate that future efforts will uncover the uncharacterized genes at the cell/organelle level and further explore their scavenging functions.

## 13. Manipulating ROS Signaling to Enhance Plant Photosynthetic Efficiency and Crop Yield

**Figure 5:**
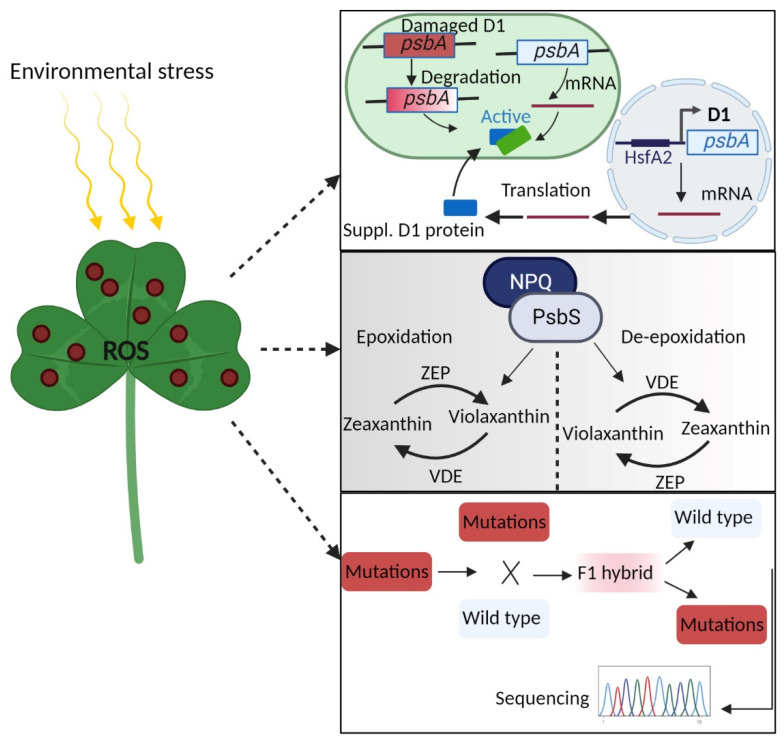
Manipulation of ROS toxicity in response to environmental cues. Excessive ROS production under extreme environment damage chloroplast-related proteins. Using bioengineering approaches, supplementing the nuclear D1 protein pathway into chloroplast and installation of xanthophyll cycle mediating reversible inter-conversion of ZEP and VDE could enhance photosynthetic efficiency. In addition, functional characterization of random mutations could help plants to adapt to undesirable environmental cues efficiently.

Plants being sessile organisms are routinely exposed to environmental fluctuations (Figure 5). Photosynthetic machinery is sensitive to changing light intensities, and photo-damage may occur in response to HL intensities. Photosynthate production (i.e., crop yield) is highly dependent on light energy conversion into chemical energy during photosynthesis [203]. Photoinhibition or damaged PSII affects the D1 protein turnover, which takes part in PSII repairing, encoded by a chloroplastic *psbA* gene [204]. The activated PSII repair process first degrades the damaged D1 proteins and then synthesizes D1 precursors (pre-D1) and D1 to reactivate PSII [205]. Recently, a bioengineering strategy was employed to install a D1 pathway in the nucleus that enhance the D1 turnover, ultimately leading to enhanced photosynthesis and plant yield under both control and high-temperature conditions [204]. Genetic engineering of nuclear D1 with plastid peptide sequence of RuBisco under the control of heat shock promoter *AtHsfA2* promotes PSII activities with tolerance against heat stress in *Arabidopsis thaliana, Nicotiana tabacum,* and *Oryza sativa*. The de novo synthesis of D1 precursors both in chloroplast and nucleus mitigates the deficient D1 demand required for repairing PSII under photo-damage [204]. 

In addition, plants have adopted photo-protective mechanisms, such as NPQ, which prevent the over-excitation of photosynthetic light-harvesting antenna complexes [134]. Although the excitation of NPQ chlorophyll fluorescence may happen rapidly, the relaxation phase is gradual [206], thus exposing the PSII to repeated, varying light intensities and low quantum yield. The slow recovery and decreased CO_2_ fixation under quenched and non-quenched states, allow the photosynthetic apparatus acclimatization to occur via thylakoid-associated protein PsbS and the xanthophyll cycle [207,208]. Under light fluctuations, the xanthophyll reactions are catalyzed by the reversible inter-converted pigment molecules, zeaxanthin and violaxanthin, which are mediated by zeaxanthin epoxidase (ZEP) and violaxanthin de-epoxidase (VDE) respectively. It was revealed that ZEP accumulation and NPQ installation show a similar mechanism in *Oryza sativa*, *Hordeum vulgare,* and *Spinacia oleracea* under varying irradiance absorbance [209]. In addition, the plants retain the potential to establish NPQ under HL and more rapid re-establishment of photosystem complexes under decreased light has also been anticipated [210,211] indicating balanced or increased plant biomass under varying light intensities. Recently, Kromdijk et al. [212] and Garcia-Molina et al. [213] employed bioengineering of xanthophyll cycle components and PsbS protein in *Nicotiana tabaccum* and *Arabidopsis thaliana*, respectively. The findings that overexpression of *VDE*, *ZEP*, and *PsbS* enhanced photo-protection in response to NPQ in both species, and promoting biomass in tobacco, demonstrating that NPQ is essential for plant growth but may interfere with decarboxylation capacity, excessive energy distribution, or retrograde signaling. Moreover, the overexpressed lines in tobacco revealed higher capacities for CO_2_ uptake, accounting for the accumulated biomass [212]. 

The core component of the xanthophyll cycle and PsbS protein with NPQ activities have been found conserved among flowering plants and green algae [214]. In the microalgae, *Chlamydomonas reinhardtii,* random mutations in *Light Response Signaling protein 1 (LRS1),* a homolog of plant phospholatory protein COP1 were found to be associated with HL adaptation [215]. Similarly, in the cyanobacteria *Synechocystis* sp., the functional characterization of representative mutant proteins revealed higher adaptation under HL cues. In contrast, a significant negative correlation was found between the overexpression of antioxidative systems and plant biomass accumulation. Such as APXs hinder the accumulation of H_2_O_2_ accumulation in the nucleus inhibiting the HL-responsive gene expression [62]. Therefore, we are still in the early stages of understanding the role of bioengineering for regulatory elements and antioxidants for increasing crop productivity. 

## 14. Concluding Remarks and Future Perspectives

ROS toxicity affects almost all aspects of plant metabolism and plants have evolved ROS scavenging both at cellular-based and antioxidant-based systems. ROS such as H_2_O_2_ and O_2_^−^ have important functions in chloroplast, mitochondria, and nuclei. Excessive ROS is either passively diffused via aquaporins or scavenged by antioxidative enzymes and antioxidants. Although several antioxidative enzymes and antioxidants have been identified in plants, a clear model for ROS toxicity at all levels and the participation of each antioxidant in response to multiple external and internal stimuli has yet to be described. Furthermore, there may exist other antioxidant members or isoenzymes of complex photosynthesis ROS regulation awaiting discovery in plants. Photosynthesis metabolism is regulated by evolutionary conserved systems, such as TRX and NTRC. Transcriptome-wide identification of redox regulators of photosynthesis has been analyzed in green algae and higher plants. Recent studies suggest that Trx and NTRC systems play important roles in maintaining photosynthetic efficiency. Therefore, the existing question of how redox systems interact with photosynthesis machinery is becoming clearer than before. However, some redox regulators and systems are still uncharacterized in plants. Considering that redox regulatory pathways have been established, we anticipate that future studies will explore these uncharacterized components at transcriptome levels and functional validation in photosynthesis. Such as, several isoforms and groups related to Trx- system, such as, *m-*, *h-*, *z-,* etc. have been identified but a few provide clear insights into the association with CBC enzyme and others’ participation in reduction processes either directly or indirectly has yet to be evaluated. In addition, redox homeostasis was shown to be regulated by evolutionarily conserved enzymes with antioxidative properties catalyzing photosynthetic efficiency and stress response. 

Distinct photosynthetic machinery has evolved similar and diverse features for regulating ROS levels within chloroplasts. In Calvin cycles, Trx and NTRC interaction with photosynthesis enzymes are clearer than C4 photosynthesis, which involves chloroplasts of mesophyll and bundle sheath cells. To further enhance understanding of these types of photosynthesis, key steps will involve the identification of redox regulatory genes, Cys residues, and their interaction with C4 and CAM photosynthetic enzymes. Recently, a few studies have characterized Trx-based regulators in the model plant Arabidopsis and their associated cellular and biological functions, while it remains elusive in other plants. In addition, antioxidant-mediated pathways regulating plant growth, development, and stress response in all types of photosynthesis will be helpful in future crop breeding programs for the enhancement of photosynthesis. 

Considerable bioengineering and fast-forward genetic approaches have been utilized to maintain protein efficiency in model plants and some staple crops, but fewer studies were conducted in key early divergent evolutionarily important green plant species and many crops. Therefore, future research focus should be placed on those plant species to improve our understanding of the molecular evolution of redox homeostasis of in photosynthetic machinery and to increase crop yield potential for future food security. 

## Supplementary Materials

The following supporting information can be downloaded at: https://www.mdpi.com/article/10.3390/antiox11112085/s1, Figure S1: Barley plant growth under low and high H_2_O_2_; Figure S2: Three-dimensional (3D) protein structures of GAPDH and TrxL2.2 in representative plant and alga species; Figure S3: Phylogenies of redox related genes in plants and algae; Figure S4: Phylogenies of photosynthesis related genes in plants and algae; Figure S5: Phylogenies of antioxidant related genes in plants and algae; Figure S6: Schematic representation of cyclic and non-cyclic electron flow. Table S1: ROS, half-life, migration distance, their production sites and known functions in Plants.
