# Supplementary material for: Molecular Regulation and Evolution of Redox Homeostasis in Photosynthetic Machinery
Source: Antioxidants (Basel). 2022 Oct 22;11(11):2085. doi: 10.3390/antiox11112085 (PMC9686623; doi:10.3390/antiox11112085)
Supplement: Supplementary file 1 [file antioxidants-11-02085-s001.zip › antioxidants-1925931-supplementary.pdf]

Supplementary Table

Table S1. ROS, half-life, migration distance, their production sites and known functions in Plants

Reactive oxygen species	Half-life	Migration distance	Production sites and known actions
$O_2^{\bullet -}$	1-4 μs	30 nm	**Sites:** Apoplast, chloroplast, mitochondria, nuclei, peroxisomes, ETC<br>**Actions:** Dismutation occur to H_2O_2, react with Fe-S proteins
$^1O_2$	1-4 μs	30 nm	**Sites:** Chloroplast, nuclei, membranes<br>**Actions:** Cause oxidation of lipids, proteins and DNA and Met/Cys residues
H_2O_2	>1 ms	>1 μm	**Sites:** Apoplast, chloroplast, mitochondria, peroxisomes, cytosol<br>**Actions:** react with Met and Cys residues and DNA
$^{\bullet}OH$	1 ns	1 nm	**Sites:** Fenton reaction<br>**Actions:** Highly reactive with DNA, RNA, protein and lipids biomolecules

Supplementary Figures

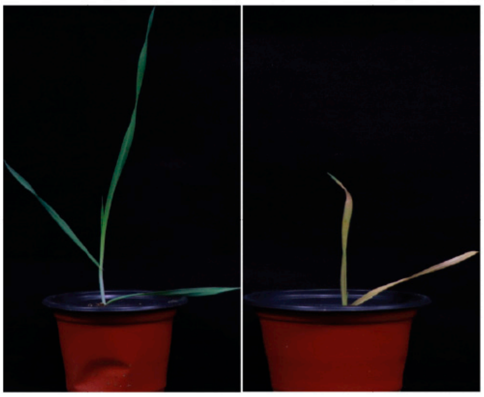

Figure S1. Barley plant growth under low and high H_2O_2

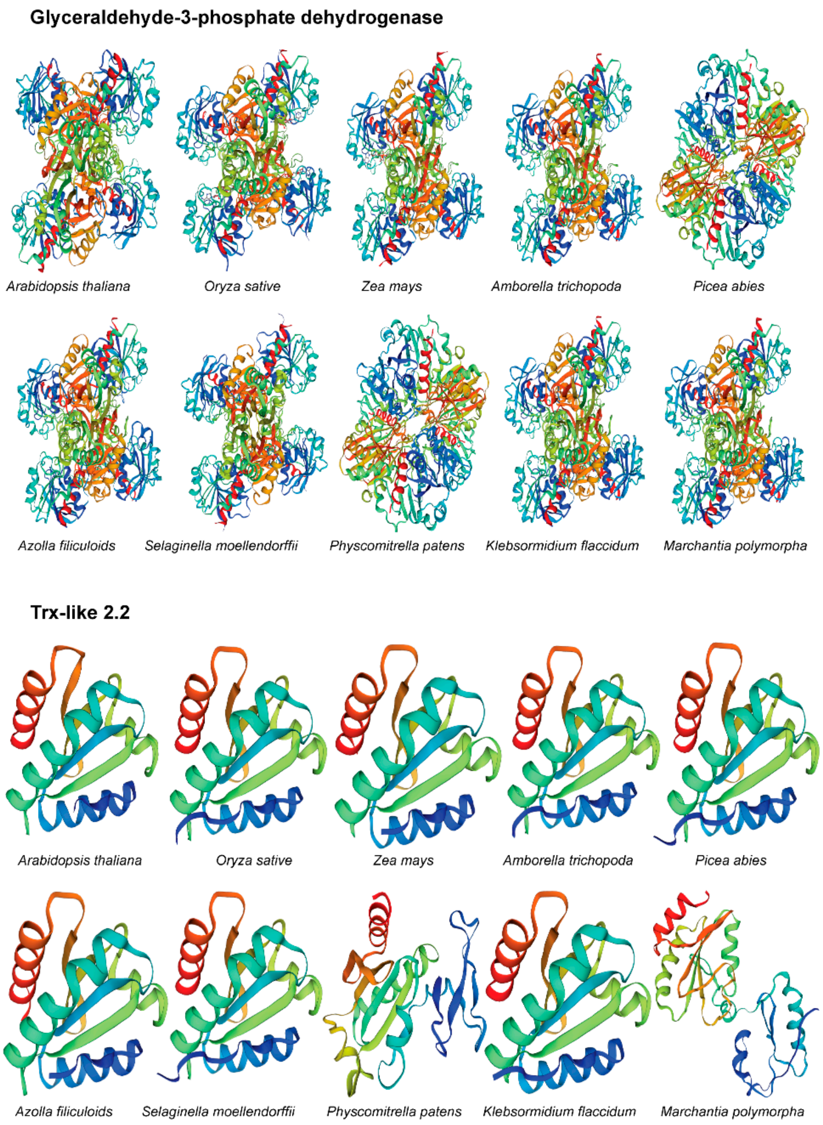

Figure S2. Three-dimensional (3D) protein structures of GAPDH and TrxL2.2 in representative alga and plant species.

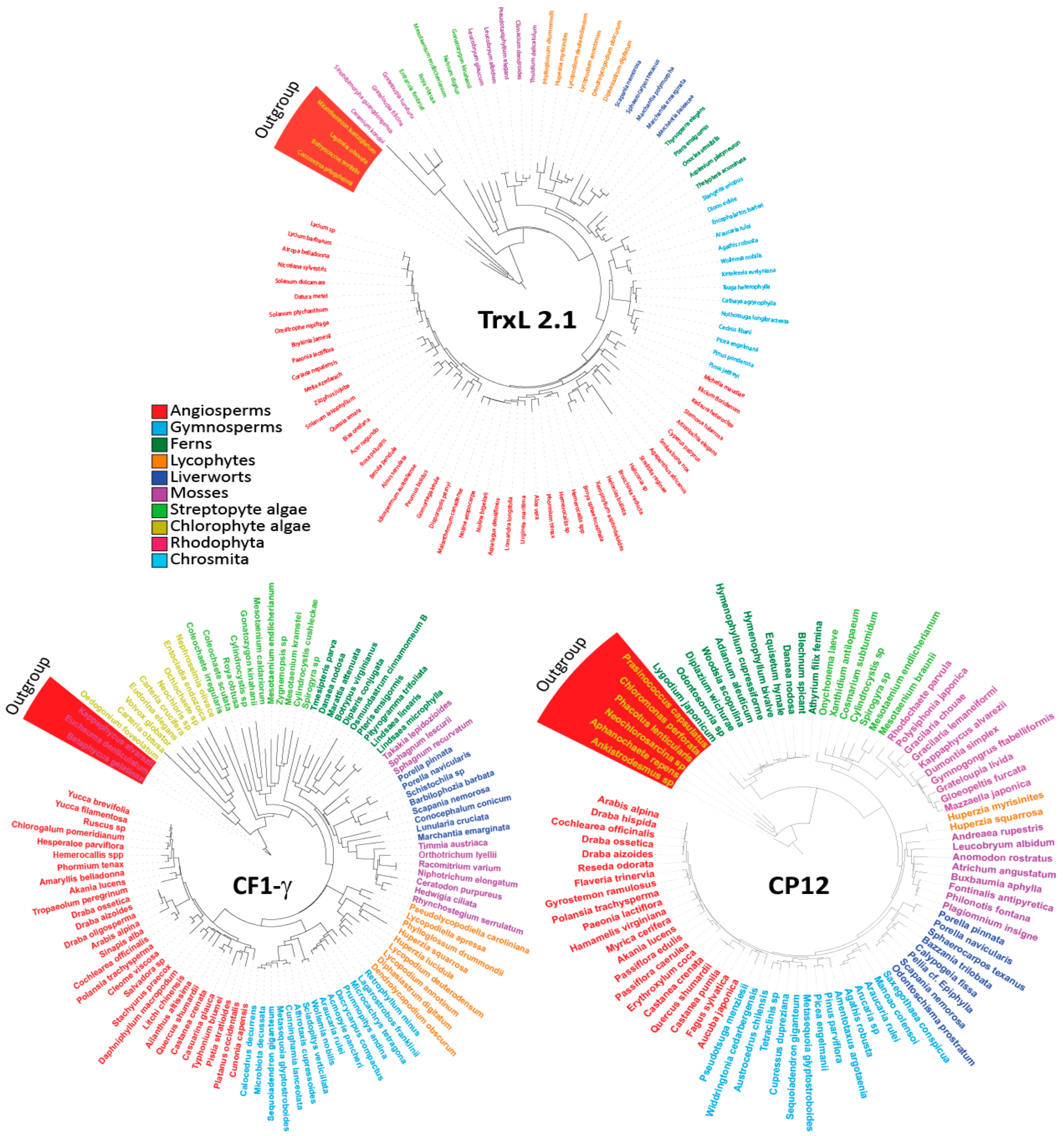

Figure S3. Phylogenies of redox related genes in plants and algae.

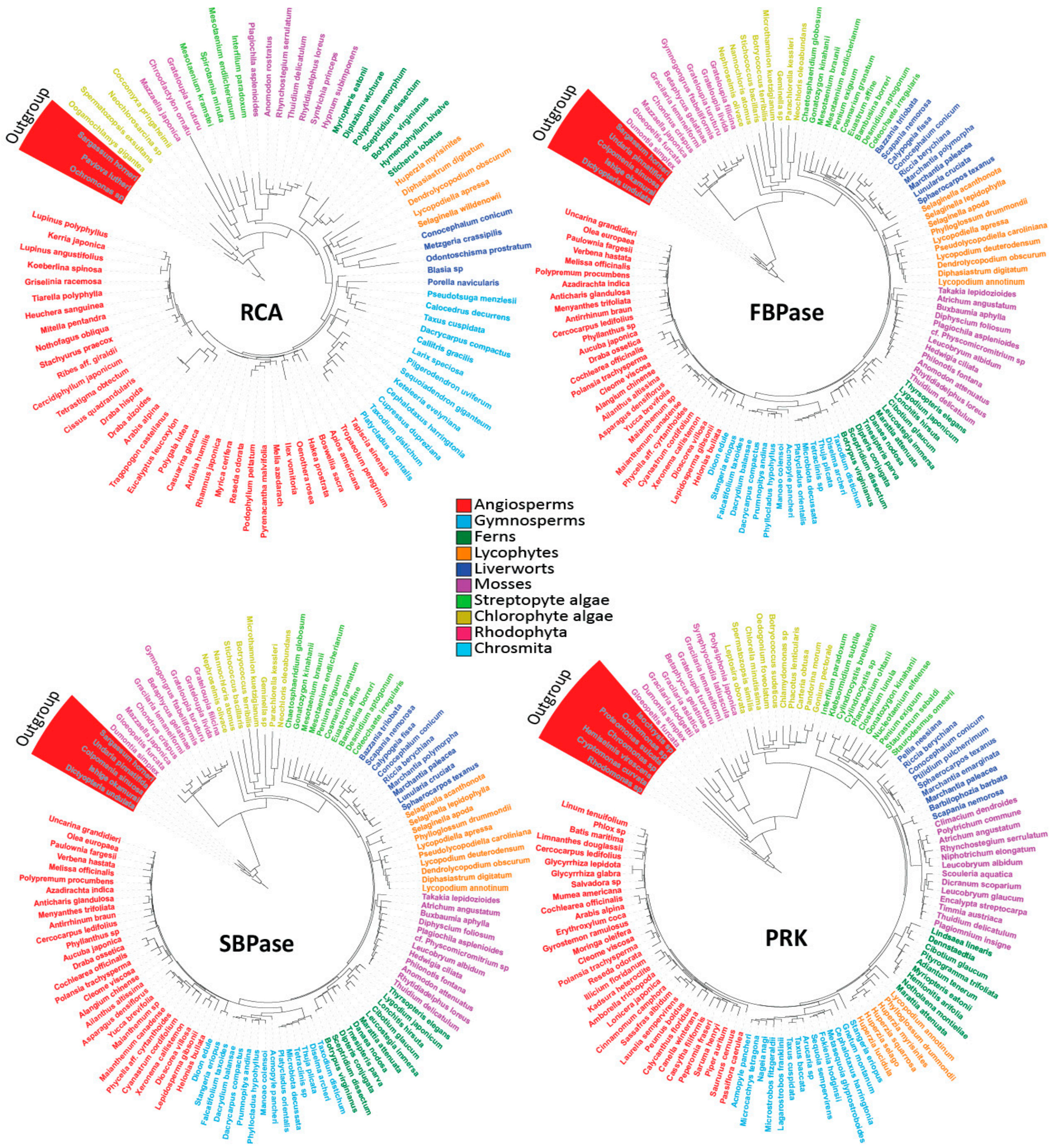

Figure S4. Phylogenies of photosynthesis related genes in plants and algae.

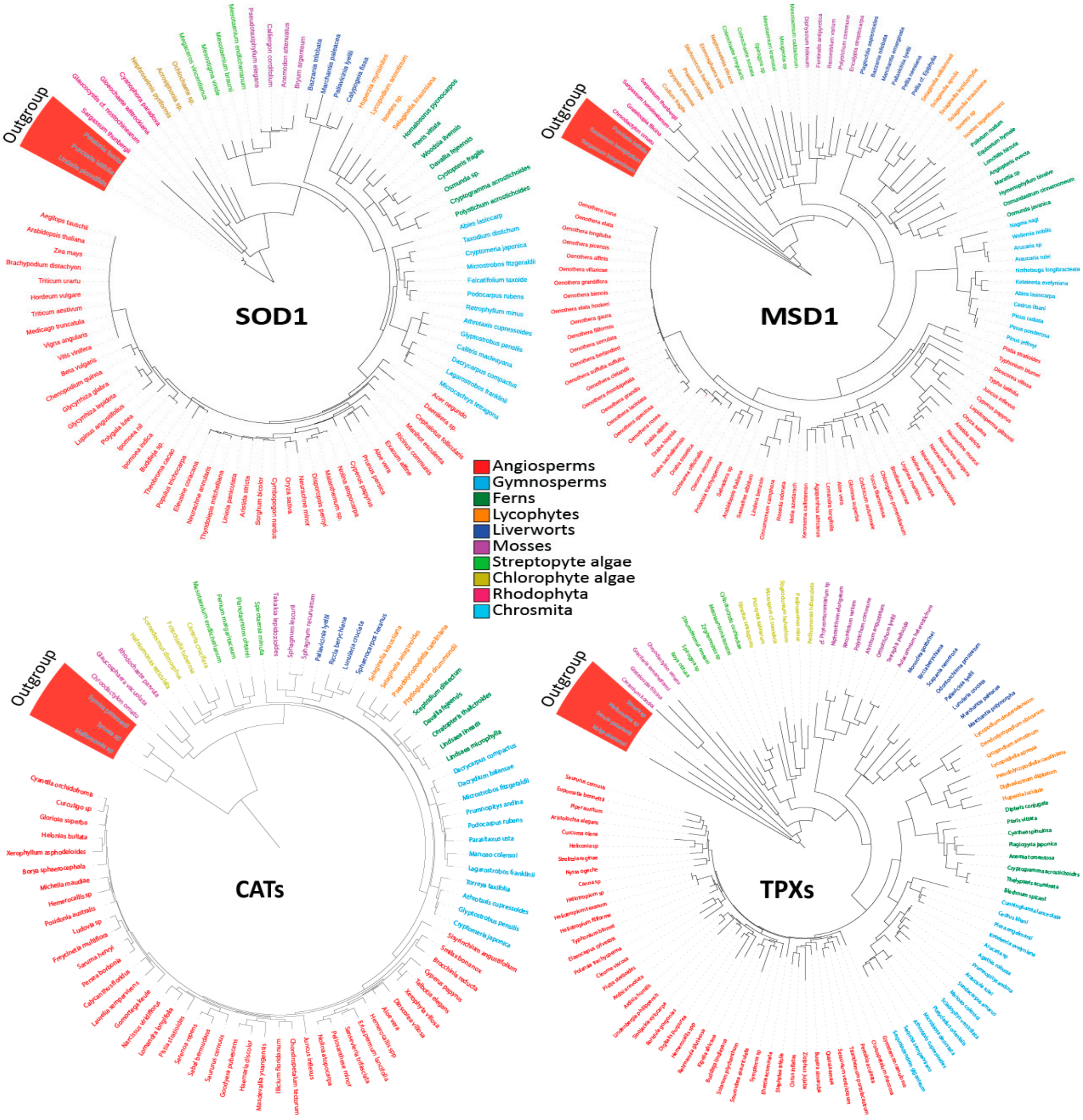

Figure S5 (A). Phylogenies of antioxidant related genes in plants and algae.

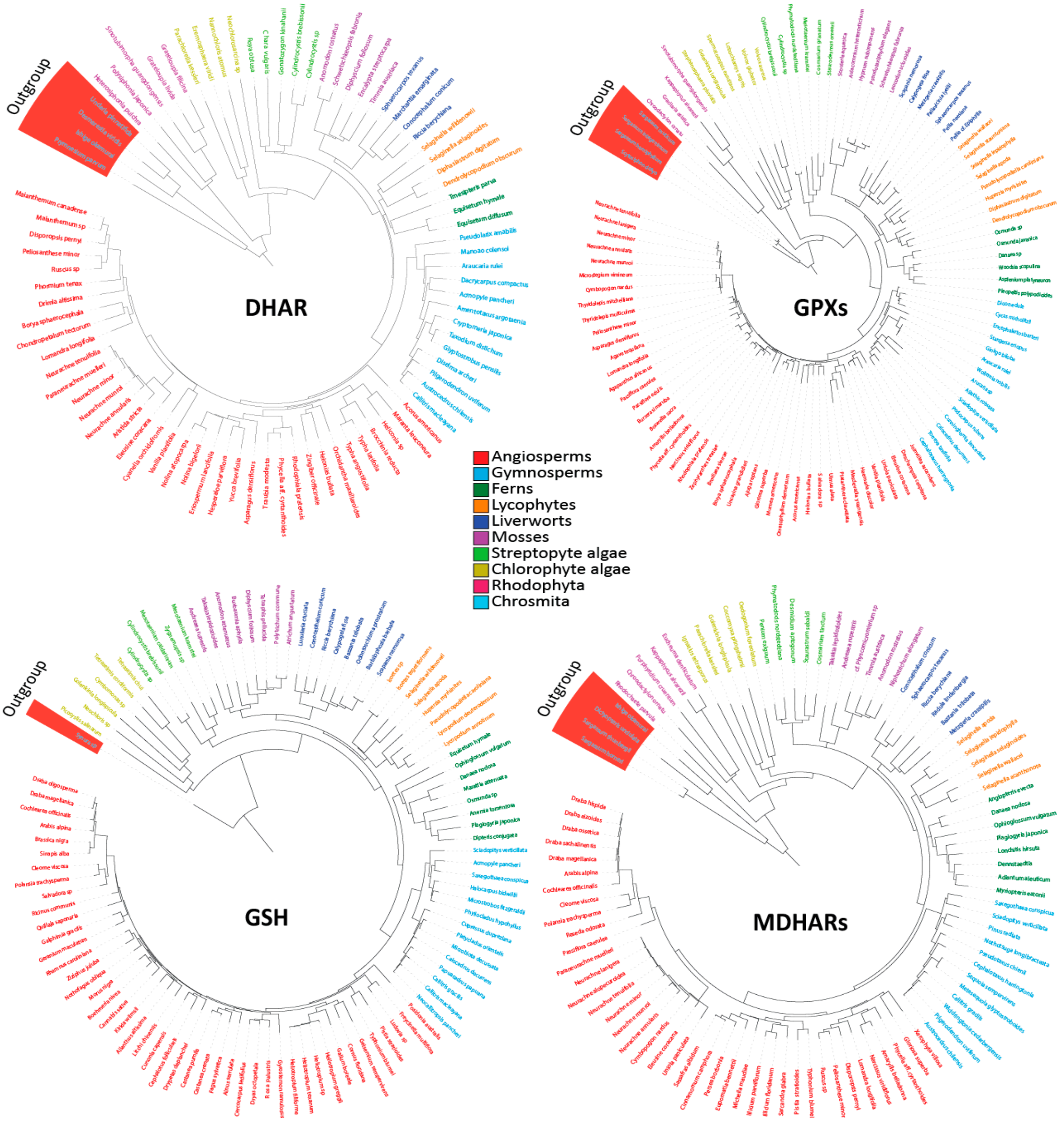

Figure S5 (B). Phylogenies of antioxidant related genes in plants and algae.

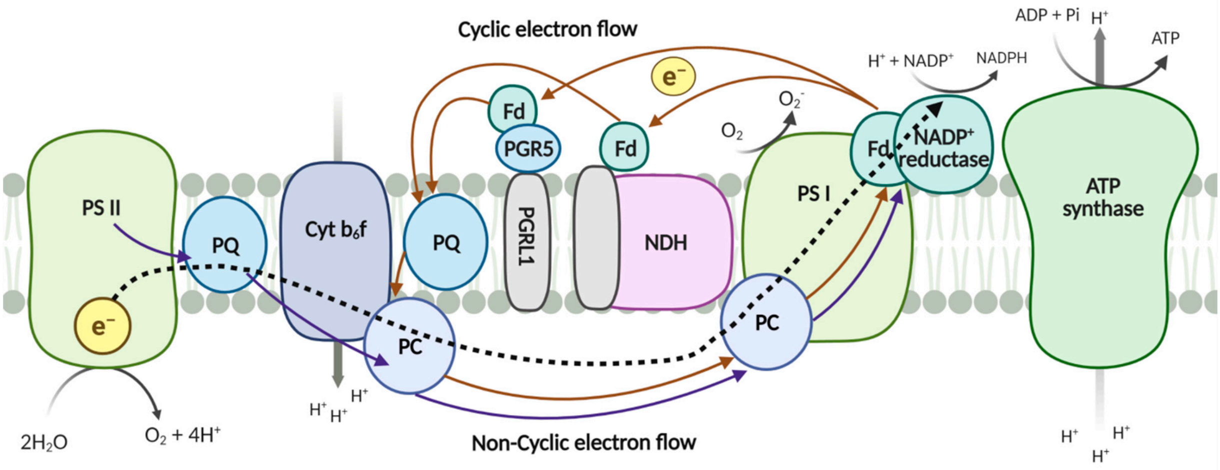

Figure S6. Schematic representation of cyclic and non-cyclic electron flow.
